# Chordopoxvirus protein F12 implicated in enveloped virion morphogenesis is an inactivated DNA polymerase

**DOI:** 10.1186/1745-6150-9-22

**Published:** 2014-11-06

**Authors:** Natalya Yutin, Guilhem Faure, Eugene V Koonin, Arcady R Mushegian

**Affiliations:** 1 National Center for Biotechnology Information, National Library of Medicine, National Institutes of Health, Bethesda, MD 20894, USA; 2 Molecular and Cellular Biosciences Division, National Science Foundation, Arlington, VA 22230, USA

**Keywords:** DNA polymerase, Exaptation, Poxviruses, Evolution of viruses

## Abstract

Through the course of their evolution, viruses with large genomes have acquired numerous host genes, most of which perform function in virus reproduction in a manner that is related to their original activities in the cells, but some are exapted for new roles. Here we report the unexpected finding that protein F12, which is conserved among the chordopoxviruses and is implicated in the morphogenesis of enveloped intracellular virions, is a derived DNA polymerase, possibly of bacteriophage origin, in which the polymerase domain and probably the exonuclease domain have been inactivated. Thus, F12 appears to present a rare example of a drastic, exaptive functional change in virus evolution.

**Reviewers:** This article was reviewed by Frank Eisenhaber and Juergen Brosius. For complete reviews, go the Reviewers’ Reports section.

## Findings

Genomes of large viruses, in addition to a small core of viral hallmark genes, encompass numerous genes that apparently have been acquired from the hosts at different stages of evolution [1-3]. Some of these genes, such as diverse metabolic, repair and signaling enzymes, retain their original biochemical activities that are utilized for virus reproduction. For other gene products, the original function is mechanistically exploited but part of the functionality has been lost during virus evolution converting the gene products into inhibitors or modulators of the respective host pathways, such as programmed cell death or various forms of immunity, a phenomenon often called molecular mimicry and especially well characterized in poxviruses [4-7]. However, several cases have been reported where the acquired host gene seems to have been exapted [8] for a function in virus reproduction that was not obviously related to the original one. For example, the poxvirus D4 protein, a uracil DNA glycosylase, functions as a processivity subunit of the viral DNA polymerase, a role for which the enzymatic activity of D4 is not required [9]. Another case in point is the poxvirus F16 protein which appears to be an inactivated serine recombinase and unexpectedly localizes to the nucleoli of the infected cells although its role in virus reproduction remains obscure [10]. We report here that poxvirus protein F12 that has been implicated in intracellular enveloped virus (IEV) morphogenesis, and in particular IEV movement along microtubules [11-13], is a derived DNA polymerase in which both the polymerase and the exonuclease activities apparently were abrogated as a result of mutational replacement of catalytic amino acid residues. This finding reveals another, striking case of exaptation in virus evolution.

### Chordopoxvirus protein F12 is an inactivated homolog of Family B DNA polymerases

In the course of a survey of the evolutionary provenance of poxvirus proteins, we unexpectedly observed that PSI-BLAST searches against the non-redundant database (NCBI, NIH, Bethesda) initiated with the amino acid sequence of Vaccinia virus (VACV) protein F12 (GenBank Accession No Q80HX6) detected, in addition to the highly significant similarity to the homologs from all chordopoxviruses, a marginal, not statistically significant similarity to several identified or putative DNA-dependent DNA polymerases (DNAPs) from plant and fungal mitochondrial plasmids and bacteriophages. To further investigate the possible homology of F12 and DNAPs, we used the sequence of the F12 homolog encoded by the most distant from VACV, early branching chordopoxvirus, the Nile Crocodile Virus (NCV) (YP_784228), as the query for a new PSI-BLAST search. This third iteration of this search identified statistically significant similarity (E-value <0.001) between F12 and a variety of organellar plasmid and phage DNAPs. Further sequence analysis was performed using the HHPred method which compares Hidden Markov Model profiles derived from the multiple alignment of readily detectable homologs of the query protein to databases of profiles of structurally characterized protein families. The HHPred search initiated with the sequence of either VACV F12 or the NCV homolog of F12 consistently yielded alignments with the *Bacillus subtilis* phage Phi29 DNAP (pdb 2py5), with a probability values greater than 98.5, which is considered strong evidence of homology, and a close correspondence between secondary structure elements (see Additional file 1). Somewhat weaker similarity was observed with a variety of plasmid-encoded DNAPs. Similar results were obtained with the Phyre2 method for protein structure prediction (see Additional file 2).

**Figure 1:**
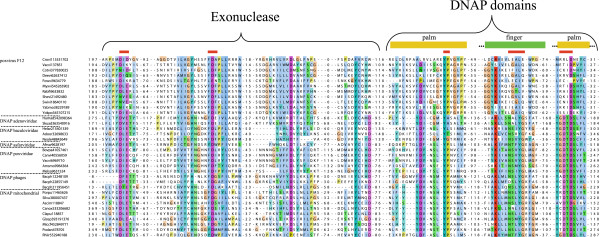
**Multiple sequence alignment of F12 proteins and family B DNAPs.** Alignment blocks containing the conserved motifs implicated in the exonuclease and polymerase activities of the DNAPs are shown, with the catalytic amino acid positions marked with red bars. The conserved blocks are separated by numbers that indicate the lengths of poorly conserved sequence segments that are not shown (see Additional file 3 for full alignment). Each sequence is denoted by the species abbreviation and GenBank Identification (GI) number. Species abbreviations: Adoor, *Adoxophyes orana* granulovirus; Afrsw, African swine fever virus; Amsmo, *Amsacta moorei* entomopoxvirus ‘L’; Ascim, *Ascobolus immersus*; Bacph, Bacillus phage; Bovpa, Bovine papular stomatitis virus; Canox, *Candida oxycetoniae*; Canvi, Canarypox virus; Clapu, *Claviceps purpurea*; Cotvi, Cotia virus SPAn232; Deevi, Deerpox virus; Fowvi, Fowlpox virus; Glosp, Glomus sp. DAOM 229456; Helar, *Helicoverpa armigera* multiple nucleopolyhedrovirus; Humad, Human mastadenovirus B; Melsa, *Melanoplus sanguinipes* entomopoxvirus; Miccf, Microbotryum cf. violaceum BFL-2013; Myxvi, Myxoma virus; Neole, *Neodiprion lecontei* nucleopolyhedrovirus; Podan, *Podospora anserina*; Porpu, *Porphyra purpurea*; Rabfi, Rabbit fibroma virus; Rhiir, *Rhizophagus irregularis* DAOM 181602; Shevi, Sheeppox virus; Silvu, *Silene vulgaris*; Skuad, Skua adenovirus 1; Swivi, Swinepox virus; Vacvi, Vaccinia virus; Yabmo, Yaba monkey tumor virus; Yokpo, Yoka poxvirus.

Taken together, these observations indicate that chordopoxvirus F12 proteins are homologs of family B DNAPs, with the strongest sequence similarity observed with the protein-primed DNAPs of phages and organellar plasmids. The family B DNAPs consist of an N-terminal 3′-5′-exonuclease (Exo) domain and the C-terminal polymerase moiety that encompasses the Palm, Fingers and Thumb domains [14,15]. The Exo and Palm domains show high level of sequence conservation throughout the family whereas the Fingers and Thumb domains are poorly conserved. Examination of the multiple alignment of the F12 proteins with the DNAPs shows that most of the amino acid residues that belong to the conserved motifs of the Palm domain and contribute to catalysis are replaced in F12 indicating that the polymerase activity has been lost in the viral proteins (Figure 1). The catalytic motifs of the Exo domain show a greater degree of conservation in F12, so the possibility that some level of exonuclease activity persists in some of the viral proteins cannot be ruled out (Figure 1).

Poxviruses encode their own, functional family B DNAPs that is essential for virus replication [6]. The PFAM families encompassing the Exo and Palm domains of these enzymes were also observed in HHPred searches but the level of similarity between F12 proteins and virus DNAPs was substantially lower than that between F12 and phage DNAPs, indicating that F12 is unlikely to have arisen via a within-genome duplication of poxvirus DNAPs.

**Figure 2:**
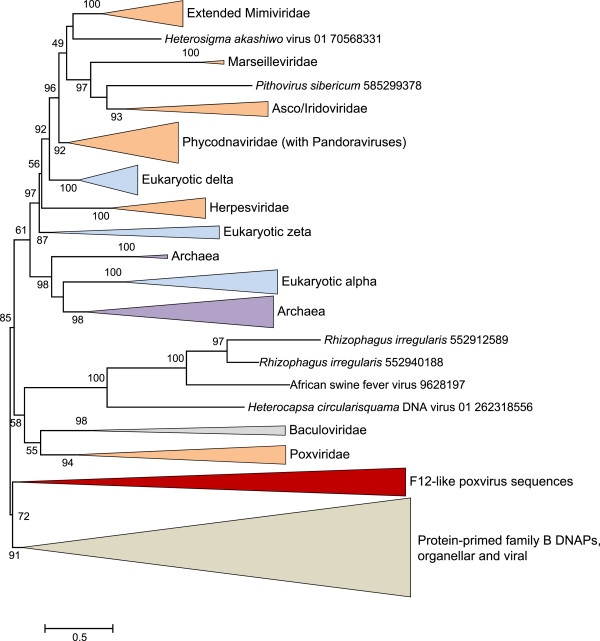
**Phylogenetic tree of the family B DNAPs including F12 proteins.** For the multiple alignment used for the phylogenetic analysis, see Additional file 3. Multiple sequences from several clades are collapsed and shown with triangles. Approximate bootstrap values calculated by FastTree are shown for each internal branch.

The multiple alignment of various DNAPs and chordopoxvirus F12 proteins (see Additional file 3) was used to infer a phylogenetic tree in which F12 clustered with the protein-primed phage and plasmid DNAPs, albeit with a moderate bootstrap support (Figure 2). This phylogeny should be interpreted with caution, especially given the acceleration of evolution of the F12 gene, likely associated with the inactivation of the enzymatic domains. Nevertheless, together with the results of sequence and structure similarity searches, these findings suggest the possibility that a bacteriophage DNAP gene was acquired by the ancestral chordopoxvirus *via* horizontal gene transfer. This acquisition was then followed by exaptation for a role in IEV morphogenesis and transport along microtubules [11] and the concomitant disruption of the DNAP catalytic centers. A notable parallel is the likely acquisition of the F16 gene, located in the same region of chordopoxvirus genomes, from a bacteriophage gene, followed by the elimination of the enzymatic (recombinase) activity, also most likely early in poxvirus evolution [10]. At least one other gene that is conserved among chordopoxviruses, G6, apparently was acquired from a bacterial source [16]. Thus, the origin of chordopoxviruses seems to have involved a substantial contribution from bacteria and their viruses.

### No relationship between F12 and the TPR repeats of kinesin light chains

The poxvirus F12 protein has been claimed to share functionally relevant similarity with the tetratricopeptide repeats (TPR) region of kinesin light chains (KLC) although no quantitative evidence has been presented in support of this connection [13]. However, no similarity to TPR repeats was detected in our search of the Conserved Domain Database at the NCBI or using the more sensitive HHPred search. More important, the presence of all-alpha TPR repeats is incompatible with the homology of F12 with the alpha-beta DNAP domains or the predicted secondary structure of F12 (Additional file 1). Identification of multiple TPRs has been reported also for two other chordopoxvirus proteins that contribute to IEV maturation and motility, namely for E2, which forms a complex with F12 [11], and for A36 [13]. Using a TPR predictor tool, we detected no TPRs in F12, E2, and A36 whereas multiple TPRs were confidently predicted in KLC (Additional file 4).

It has been reported that F12 is required for the recruitment of kinesin-1 which enables the movement of IEV along microtubules in VACV-infected cells and that deletion of the purported TPRs in F12 abrogated kinesin binding [13]. The findings described here do not conflict with these experimental observations but suggest that the interaction between F12 and kinesin is mediated by the derived DNAP domains that are unrelated to TPRs.

## Conclusions

We report here the unexpected finding that a chordopoxvirus protein implicated in IEV morphogenesis and intracellular motility is a derived, inactivated DNAP. The results of sequence comparison and phylogenetic analysis suggest an evolutionary scenario in which the F12 gene evolved from an acquired bacteriophage DNAP gene, with both exonuclease and polymerase activities apparently abrogated as indicated by the disruption of the respective catalytic sites. Alternative routes of evolution, such as duplication and subsequent inactivation of the ancestral poxvirus DNAP, cannot be formally ruled out but appear much less likely. Inactivated DNAPs has been described previously in archaea and eukaryotes [15,17,18], and recently, it has been shown that the UL8 subunit of herpes virus DNA primase is an inactivated family B DNAP [19]. However, in all these cases, the inactivated polymerases still function in DNA replication or repair, conceivably interacting with some of the same partners as active DNAPs do. The poxvirus F12 seems to present the case where an inactivated DNAP is exapted for a completely new role in a different cellular location. Characterization of potential structural features linking the complexes of F12 with its protein partners and possibly with replication complexes could be an intriguing experimental task.

## Methods

### Sequence analysis and phylogenetic tree construction

The non-redundant database of protein sequences at the NCBI was searched using the PSI-BLAST program [20]. Protein sequences were aligned using MUSCLE [21]; gapped columns (more than 30% of gaps) and columns with low information content were removed from the alignment [22]. For the purpose of visualization, alignment columns were colored using Jalview [23], with the ClustalX coloring conventions [24] and conservation color increment set at 10. Profile-against-profile searches were performed using the HHPred method [25]. Protein structure prediction was performed using the Phyre2 software [26]. Phylogenetic analysis was performed using the FastTree program with default parameters (JTT evolutionary model, discrete gamma model with 20 rate categories) [27]. The TPRs were predicted using the TPRpred software [28].

## Reviewers’ comments

### Reviewer 1: Frank Eisenhaber, Bioinformatics Institute, Singapore

The proposed MS describes the discovery of chordopoxvirus F12 phylogenetic history as a inactivated DNA polymerase. The MS is well written, logically rigorous and worth being published.

Authors’ response: *We appreciate this laconic yet encouraging review*.

### Reviewer 2: Juergen Brosius, University of Muenster

The finding that chordopoxvirus protein F12 is an acquisition of a DNA polymerase of likely bacterial origin and a subsequent exaptation as a protein with different viral function(s) is very interesting.

Unlike other examples of DNA polymerases (DNAPs) that lost their central function but still are involved in DNA replication or repair, the F12 protein has been recruited (fortuitously) into an entirely different viral task, namely intracellular virus morphogenesis and transport. As an aside, the term inactivated DNAPs is somewhat imprecise and misleading as they still exhibit a function. Perhaps, partially inactivated would be a better term.

Authors’ response: *The term “inactivated” pertains to enzymatic activities not to the loss of any functionality. We thought this should be clear but in order to make the text unequivocal in this regard, we modified the penultimate sentence of the introductory paragraph as follows*:

“We report here that poxvirus protein F12 that has been implicated in intracellular enveloped virus (IEV) morphogenesis, and in particular IEV movement along microtubules [11-13], is a derived DNA polymerase in which both the polymerase and the exonuclease activities apparently were abrogated as a result of mutational replacement of catalytic amino acid residues.” *Language to the same effect was also inserted into the Conclusions*.

It is not clear whether any of the bona fide family B DNAPs of chordopoxviruses are shown in any of the alignments, although it is stated that there is most likely no relationship to invoke a viral gene duplication event.

Authors’ response: *The alignment in* Figure 1*actually includes 5 poxvirus DNAP sequences that are explicitly marked as such. The problem probably is with the truncation of the figure in the original pdf file. We are taking care of this in the revision*.

The term horizontal transfer is missing from the text. Does it mean that acquisition of an originally bacteriophage DNAP gene by a virus infecting eukaryotic cells cannot be considered HGT?

Authors’ response: *We agree that horizontal gene transfer is an appropriate term to describe this case and include it in the revised text*.In the pdf version of the manuscript, Figure 1 is truncated. Among other things, the important subdivisions on the left margins can only be guessed.

Authors’ response: *We regret the inconvenience caused by poor formatting of the figure. We include a version with wider margins which should eliminate the problem*.

## Abbreviations

DNAP: DNA polymerase; Exo: Exonuclease; IEV: Intracellular enveloped virus; KLC: Kinesin light chain; NCV: Nile crocodile virus; TPR: TetratricoPeptide repeat; VACV: Vaccinia virus.

## Competing interests

The authors declare that they have no competing interests.

## Authors’ contributions

ARM made the original observation and incepted the study; NY, GF and EVK performed data analysis; EVK wrote the manuscript that was read and approved by all authors. All authors read and approved the final manuscript.

## Authors’ information

Arcady R Mushegian: The views expressed in this article are those of the author in his personal capacity and do not necessarily represent the view of the National Science Foundation or the Government of the United States.

## Supplementary Material

Additional file 1 The HHPred output for the F12 homolog of CNV. Click here for file

Additional file 2 Phyre 2 results for VACV F12. Click here for file

Additional file 3 **Multiple alignment used for the construction of the phylogenetic tree in Figure** 2**.** Click here for file

Additional file 4 TPRpred results for the relevant poxvirus proteins and kinesin light chains. Click here for file
