# Supplementary material for: Chordopoxvirus protein F12 implicated in enveloped virion morphogenesis is an inactivated DNA polymerase
Source: Biol Direct. 2014 Nov 6;9:22. doi: 10.1186/1745-6150-9-22 (PMC4304020; doi:10.1186/1745-6150-9-22)
Supplement: Additional file 1 — The HHPred output for the F12 homolog of CNV. [file 1745-6150-9-22-S1.docx]

The HHPred output for the F12 homolog of CNV

>[2py5_A](http://pdb.rcsb.org/pdb/explore.do?structureId=2PY5) DNA polymerase; protein-DNA complex, replication, transferase/DNA complex; HET: DNA; 1.60A {Bacillus

phage PHI29} SCOP: [c.55.3.5](http://scop.mrc-lmb.cam.ac.uk/scop/search.cgi?key=c.55.3.5) [e.8.1.1](http://scop.mrc-lmb.cam.ac.uk/scop/search.cgi?key=e.8.1.1) PDB:  [1xhz _A*](http://pdb.rcsb.org/pdb/explore.do?structureId=1xhz)  [1xhx _A*](http://pdb.rcsb.org/pdb/explore.do?structureId=1xhx)  [2ex3 _A*](http://pdb.rcsb.org/pdb/explore.do?structureId=2ex3)  [1xi1 _A*](http://pdb.rcsb.org/pdb/explore.do?structureId=1xi1)  [2pyj _A*](http://pdb.rcsb.org/pdb/explore.do?structureId=2pyj)  [2pyl _A*](http://pdb.rcsb.org/pdb/explore.do?structureId=2pyl)  [2pzs _A*](http://pdb.rcsb.org/pdb/explore.do?structureId=2pzs)

Probab=98.66 E-value=4.2e-06 Score=95.00 Aligned_cols=358 Identities=11% Similarity=0.081 Sum_probs=0.0

Q ss_pred cchhcccccHHHhhchhcccccCcccccccceeEEEEEEEEecCCCCceeEEEecCCceEEcCCCHHHHHHHHHHHHHHH

Q Fri_Aug_01_23: 192 PEIDVLPFDIKYISRDELWARISSSLDQTHIKTIAVSVYGAITDNGPMPYMISTYPGNTFVNFNSVKNLILNFLDWIKDI 271 (635)

Q Consensus 192 PEiDil~~Di~~ia~~~~W~~~~~~~~~~~~~~~~v~v~a~it~~gp~~ymIstyPG~~F~nf~s~~~lI~~FL~Wl~e~ 271 (635)

+...++.+|||.. + ++....++.|.+.. .+++..++. ++++.+|++||++.

T Consensus 4 ~~~r~l~fDIEt~----------~--~~~~~~i~~I~~~~---~~~~~~~~~--------------~~lL~~F~~~i~~~ 54 (575)

T 2py5_A 4 MPRKMYSCAFETT----------T--KVEDCRVWAYGYMN---IEDHSEYKI--------------GNSLDEFMAWVLKV 54 (575)

T ss_dssp -CCCEEEEEEEEC----------C--BTTBCCEEEEEEEE---SSCTTCEEE--------------ESCHHHHHHHHHHH

T ss_pred CcceEEEEEEEEE----------e--cCCCCcEEEEEEEE---CCcceEEEe--------------hhHHHHHHHHHHHc

Q ss_pred hcCCceEEEEEhhhhccchHHHHhhccccCC--ccc-cCc----eEEeCCC----------------cEEEEEEhhhhcc

Q Fri_Aug_01_23: 272 MTSTRTIILVGYMSNLFDIPLLTVYWPNNCG--WKI-YNN----TLISSDG----------------ARVIWMDAYKFSC 328 (635)

Q Consensus 272 m~n~~ti~LvGy~Ss~FD~pLL~a~wp~~~G--W~~-gn~----~lvs~~G----------------~kv~l~D~a~Fs~ 328 (635)

...+++ |+.-||+|.|....-.. | |.. |-. ..+.++| ..+.++|..++.

T Consensus 55 -----dpdii~-~N~~FD~pyL~~R~~~~-~i~~~~~g~p~~~~~~~~r~g~~~~~~~~~~g~~~~~~~~d~~D~~~l~- 126 (575)

T 2py5_A 55 -----QADLYF-HNLKFAGAFIINWLERN-GFKWSADGLPNTYNTIISRMGQWYMIDICLGYKGKRKIHTVIYDSLKKL- 126 (575)

T ss_dssp -----CCEEEE-TTHHHHHHHHHHHHHHT-TCEECTTCCTTEEEEEEBTTCCEEEEEEEEEEETTEEEEEEEEEHHHHS-

T ss_pred -----CCEEEE-EchhhcHHHHHHHHHhc-CCCcccccCccccccccccCCEEEEEEEEEccCCccceEEEEEEecccC-

Q ss_pred cCcHHHHHHhh--ccCCCCCchhhhhhhHHHHHHHHHHHHHHHHHHHHHHHHHHHHHHHHHHhCccchhhcCCHHHHHHH

Q Fri_Aug_01_23: 329 GLSLQDYCYHW--GSKPESRPFDLIKKSDAKRNTKSLVKESMASLKSLYEAFETQSGALEVLMSPCRMFSFSRIEDMFLT 406 (635)

Q Consensus 329 ~~~~~~Yc~~W--~~~~~~~p~D~i~~~e~k~~~k~l~k~s~~~~~~L~~A~~~q~~~L~~i~~p~~~~~F~~leDm~l~ 406 (635)

..+|..-+++- ++...++|.+.+.+.+.......++ +++.||.........-+-.....+.-+++.=.+..

T Consensus 127 s~sL~~va~~~gl~~~K~d~~~~~i~~~~~~~~~~l~~-------Yc~~Dv~l~~~L~~kl~~~~~~~~t~~g~~~~~f~ 199 (575)

T 2py5_A 127 PFPVKKIAKDFKLTVLKGDIDYHKERPVGYKITPEEYA-------YIKNDIQIIAEALLIQFKQGLDRMTAGSDSLKGFK 199 (575)

T ss_dssp CSCHHHHHHHTTCCCCSSCCCTTSCCCTTCCCCHHHHH-------HHHHHHHHHHHHHHHHHHTTCCSSSHHHHHHHHHH

T ss_pred CcCHHHHHHHhCCCCcccccCccccCcccchHHHHHHH-------HHHHHHHHHHHHHHHHHhhhcccccchHHHHHHHH

Q ss_pred HHHHHhhhccCCceeccCCCcHHHHHHHhh---hcceeec--CC-Cc-eeEEEeccHHHHHhcC-CCCCCCceeeccC--

Q Fri_Aug_01_23: 407 SVINRVSENTGMGMYYPTNDIPSLFIESSI---CLDYIIV--NN-QE-SNKYRIKSVLDIISSK-QYPAGRPNYVKNG-- 476 (635)

Q Consensus 407 ~~~~~~a~~~~~~~yyP~~~~a~~fI~~sI---~v~~~~i--~~-~k-~~~~~lkSvl~vi~~~-~YPvG~P~yv~~~-- 476 (635)

+.-...|-....+...|. ..++++++. +|..... .. .+ -..|+++|.-+-++.. .+|.|.|..++..

T Consensus 200 ~~~l~~~~~~~~p~~~~~---~~~~~~~~y~GG~v~~~P~~~~~~~~~i~~~D~~SLYPsim~~~~~p~g~~~~~~~~~~ 276 (575)

T 2py5_A 200 DIITTKKFKKVFPTLSLG---LDKEVRYAYRGGFTWLNDRFKEKEIGEGMVFDVNSLYPAQMYSRLLPYGEPIVFEGKYV 276 (575)

T ss_dssp HHHCHHHHHHHSCCCCHH---HHHHHHTTCCCCCEEECGGGBTCEECSEEEEEETTHHHHHHHHSCEEEEEEEEEESSCC

T ss_pred HHhhhhhhhhcccCCChh---HHHHHHHcCCCccccccccccccccceEEEEecccchHHHhccCCCCCCCcEecccccc

Q ss_pred --CCCceEE--EEEEEEecCCCcCceEecCCC-----------CceeEEEEE-eee--EHHHHHhCCCeEEEEEEEecCC

Q Fri_Aug_01_23: 477 --TKGKLYI--ALCKVTVPTNDHIPVVYHDDD-----------NTTTFITVL-TSV--DIETAIRAGYSIVELGALQWDN 538 (635)

Q Consensus 477 --~~gkLyI--ALCkVt~~~dvkiPvl~~~~d-----------~~~~F~~vL-TSV--DI~~A~rgGYkIk~lgaLeW~~ 538 (635)

++..+.+ .-|+|+ +++.+.|+|=.... .+.+|.++. |++ |+-+|...||+|+.+....|++

T Consensus 277 ~~~~~~~~~~~v~~~v~-p~~~~~p~lp~~~~l~fp~c~~~~~~~~~~~g~~~t~~~lel~~a~~~G~~i~~~~g~~f~~ 355 (575)

T 2py5_A 277 WDEDYPLHIQHIRCEFE-LKEGYIPTIQIKRSRFYKGNEYLKSSGGEIADLWLSNVDLELMKEHYDLYNVEYISGLKFKA 355 (575)

T ss_dssp CCTTSCEEEEEEEEEEE-ECTTCCCCCCC-------CCSCCSBCTTSCEEEEEEHHHHHHHHHHEEEEEEEEEEEEEEEE

T ss_pred cccCCCcccccEEEEEE-cCccCCCcccCcCcccCcccccccccCCeeEEEEecCccHHHHHHHHCCcEEEEeeEEEECC

Q ss_pred cchhhhhcchhhHHHHHHhhhcchHHHHHHHHh-------cccCCCccch--------------------------hHHH

Q Fri_Aug_01_23: 539 NIPELKNGLLDSIKMIYDLNAVTTNNLLEQLIE-------NINFNNSSII--------------------------SLFY 585 (635)

Q Consensus 539 si~i~~~~~~~~i~~l~~~~~~~t~~Ll~~~~~-------~~~~p~~~~~--------------------------l~f~ 585 (635)

...+++.= |+++++.-.-.++.+ +...+ |..+.+.... -.++

T Consensus 356 ~~~~~~~~----i~~l~~~R~~~k~~~-~~~~Kl~~NS~YG~~g~~~~~~~~~~~~~~~~~~~~~~~~~~~~~p~~~~ia 430 (575)

T 2py5_A 356 TTGLFKDF----IDKWTYIKTTSEGAI-KQLAKLMLNSLYGKFASNPDVTGKVPYLKENGALGFRLGEEETKDPVYTPMG 430 (575)

T ss_dssp ESCTTHHH----HHHHHHHHHHCCHHH-HHHHHHHHHSSHHHHTCCSBCCBEEEEECTTSSEEEEECCCCBCCCCCHHHH

T ss_pred CCChhHHH----HHHHHHHhhhcCHHH-HHHHHHHHhhhHhhhccCcccceeEEEecCcceEEEecCcccccCCcchhhh

Q ss_pred HHHHHHHHHHHHHHHHH

Q Fri_Aug_01_23: 586 TFAISYCRAFIYSIMET 602 (635)

Q Consensus 586 AFa~SYCR~~iH~LI~~ 602 (635)

++.-+|.|..+++.++.

T Consensus 431 ~~it~~~R~~l~~~~~~ 447 (575)

T 2py5_A 431 VFITAWARYTTITAAQA 447 (575)

T ss_dssp HHHHHHHHHHHHHHHHH

T ss_pred hHHHHhHHHHHHHHHHh

>[2py5_A](http://pdb.rcsb.org/pdb/explore.do?structureId=2PY5) DNA polymerase; protein-DNA complex, replication, transferase/DNA complex; HET: DNA; 1.60A {Bacillus

phage PHI29} SCOP: [c.55.3.5](http://scop.mrc-lmb.cam.ac.uk/scop/search.cgi?key=c.55.3.5) [e.8.1.1](http://scop.mrc-lmb.cam.ac.uk/scop/search.cgi?key=e.8.1.1) PDB:  [1xhz _A*](http://pdb.rcsb.org/pdb/explore.do?structureId=1xhz)  [1xhx _A*](http://pdb.rcsb.org/pdb/explore.do?structureId=1xhx)  [2ex3 _A*](http://pdb.rcsb.org/pdb/explore.do?structureId=2ex3)  [1xi1 _A*](http://pdb.rcsb.org/pdb/explore.do?structureId=1xi1)  [2pyj _A*](http://pdb.rcsb.org/pdb/explore.do?structureId=2pyj)  [2pyl _A*](http://pdb.rcsb.org/pdb/explore.do?structureId=2pyl)  [2pzs _A*](http://pdb.rcsb.org/pdb/explore.do?structureId=2pzs)

Probab=98.51 E-value=7.4e-05 Score=83.87 Aligned_cols=354 Identities=11% Similarity=0.128 Sum_probs=0.0

Q ss_pred ceEEEEecCCC--ceEEeCCCHHHHHHHHHHHHHHHHhcccccccccccccccccccccCCCeEEEEEhhhhccchHHHH

Q Fri_Aug_01_22: 245 EPVAVATYPGG--RAYFDSSCGKRVTEFLLECLAERFDAEDRRREEDGDDEPETGGAAAGGDTVILAGYHSSFFDAPLLR 322 (688)

Q Consensus 245 siymIstYP~G--~~F~n~~s~k~li~~FL~WL~E~m~~~~~~~~~~~~~~~~~~~~~~~~~TIiLvGy~SS~FD~pLLr 322 (688)

.++||+.+-+| ..|.. ++++.+|++||.+. +-.+++ |=. -||+|.|.

T Consensus 23 ~i~~I~~~~~~~~~~~~~----~~lL~~F~~~i~~~-------------------------dpdii~-~N~-~FD~pyL~ 71 (575)

T 2py5_A 23 RVWAYGYMNIEDHSEYKI----GNSLDEFMAWVLKV-------------------------QADLYF-HNL-KFAGAFII 71 (575)

T ss_dssp CEEEEEEEESSCTTCEEE----ESCHHHHHHHHHHH-------------------------CCEEEE-TTH-HHHHHHHH

T ss_pred cEEEEEEEECCcceEEEe----hhHHHHHHHHHHHc-------------------------CCEEEE-Ech-hhcHHHHH

Q ss_pred hhcCCCC-----------ccccccccccceEEECCC----------------cEEEEEEhhhhcccCCHHHHHHhh--cc

Q Fri_Aug_01_22: 323 RRASDGR-----------WVAAVGDDAGPTLIYRGR----------------HRVLVRDLGLFNPSFSPDAFVRCW--AR 373 (688)

Q Consensus 323 a~wp~~G-----------W~~~~~~~~gn~LVs~~G----------------~rvil~D~a~Fs~~~s~~~Yc~~W--~~ 373 (688)

.++...| | +..+.++| ..+.++|..++. +.+++.-+++- .+

T Consensus 72 ~R~~~~~i~~~~~g~p~~~---------~~~~~r~g~~~~~~~~~~g~~~~~~~~d~~D~~~l~-s~sL~~va~~~gl~~ 141 (575)

T 2py5_A 72 NWLERNGFKWSADGLPNTY---------NTIISRMGQWYMIDICLGYKGKRKIHTVIYDSLKKL-PFPVKKIAKDFKLTV 141 (575)

T ss_dssp HHHHHTTCEECTTCCTTEE---------EEEEBTTCCEEEEEEEEEEETTEEEEEEEEEHHHHS-CSCHHHHHHHTTCCC

T ss_pred HHHHhcCCCcccccCcccc---------ccccccCCEEEEEEEEEccCCccceEEEEEEecccC-CcCHHHHHHHhCCCC

Q ss_pred CCcCCCccccccch---HHHHHHHHHHHHHHHHHHHHHHHHHHHHHHHHHHHhCCCCCCcchhhcccHHHHHHHHHHhhc

Q Fri_Aug_01_22: 374 AERGRLDDRYLRTR---ADLERYRDQILAYLSESCYYLHCAALAQSAALAEAFGRPDPLRCPSLFDAFYEHLIARAAQVP 450 (688)

Q Consensus 374 ~~~~~p~D~~lit~---~e~~~~ik~~~k~~~~~~~~L~~A~~aq~~aL~~if~~~~~p~~~~~F~~leDm~i~~aa~~~ 450 (688)

....+|-+ .++. ++.++-++ +...=|..++.-... .+..++.+. +-.-.+...+...++-.+.+.-

T Consensus 142 ~K~d~~~~--~i~~~~~~~~~~l~~----Yc~~Dv~l~~~L~~k---l~~~~~~~~--t~~g~~~~~f~~~~l~~~~~~~ 210 (575)

T 2py5_A 142 LKGDIDYH--KERPVGYKITPEEYA----YIKNDIQIIAEALLI---QFKQGLDRM--TAGSDSLKGFKDIITTKKFKKV 210 (575)

T ss_dssp CSSCCCTT--SCCCTTCCCCHHHHH----HHHHHHHHHHHHHHH---HHHTTCCSS--SHHHHHHHHHHHHHCHHHHHHH

T ss_pred cccccCcc--ccCcccchHHHHHHH----HHHHHHHHHHHHHHH---HHhhhcccc--cchHHHHHHHHHHhhhhhhhhc

Q ss_pred cCCCcCCCCchHHHHHHHHHhhhccccceeec--cc--cceeEEEechHHHHHHHHhC--CCCCceEecCC----CCCCc

Q Fri_Aug_01_22: 451 ALAPTRCSHPDLADLLERAARRDGARVQVEGA--YT--AAERELDLRPAALRVLAREY--PVGYPYCTAAP----DLAGR 520 (688)

Q Consensus 451 A~~~yyP~~~da~~fI~~sIr~~~~~V~t~~~--~~--k~~~~~~LkS~vl~via~~Y--PvG~Pyyv~~~----~~~gk 520 (688)

.. .| .++..+++.++. .+++|..... .. +.-..|++.| .-+-++..| |.|.|..+... + +..

T Consensus 211 ~p---~~-~~~~~~~~~~~y--~GG~v~~~P~~~~~~~~~i~~~D~~S-LYPsim~~~~~p~g~~~~~~~~~~~~~-~~~ 282 (575)

T 2py5_A 211 FP---TL-SLGLDKEVRYAY--RGGFTWLNDRFKEKEIGEGMVFDVNS-LYPAQMYSRLLPYGEPIVFEGKYVWDE-DYP 282 (575)

T ss_dssp SC---CC-CHHHHHHHHTTC--CCCCEEECGGGBTCEECSEEEEEETT-HHHHHHHHSCEEEEEEEEEESSCCCCT-TSC

T ss_pred cc---CC-ChhHHHHHHHcC--CCccccccccccccccceEEEEeccc-chHHHhccCCCCCCCcEeccccccccc-CCC

Q ss_pred EEE--EEEEEEecCCccCceEEcC---------------C-ceeEe-eeeEHHHHHHh-CCceEEEEEEEecCCcchhhH

Q Fri_Aug_01_22: 521 LSL--VRCRVEAKPQNRFPVLAAD---------------G-GEGVY-TSAEVEYAVRV-LDCRVEVLEALEWPGRAPIFR 580 (688)

Q Consensus 521 LyI--ALCeVt~k~dvkiPVL~~d---------------~-F~~vL-TSVDIe~Avrl-gGYkIkvl~ALEW~~si~I~~ 580 (688)

+.+ ..|+|+ +++.++|+|-.. + |.+++ |++|||+...+ .||+|+++....|+....+|.

T Consensus 283 ~~~~~v~~~v~-p~~~~~p~lp~~~~l~fp~c~~~~~~~~~~~g~~~t~~~lel~~a~~~G~~i~~~~g~~f~~~~~~~~ 361 (575)

T 2py5_A 283 LHIQHIRCEFE-LKEGYIPTIQIKRSRFYKGNEYLKSSGGEIADLWLSNVDLELMKEHYDLYNVEYISGLKFKATTGLFK 361 (575)

T ss_dssp EEEEEEEEEEE-ECTTCCCCCCC-------CCSCCSBCTTSCEEEEEEHHHHHHHHHHEEEEEEEEEEEEEEEEESCTTH

T ss_pred cccccEEEEEE-cCccCCCcccCcCcccCcccccccccCCeeEEEEecCccHHHHHHHHCCcEEEEeeEEEECCCCChhH

Q ss_pred hHHHHHHHhhhccccccchhhhHHHHHHhcCh-hhhhcccccchhhc----------------------CchhHHHHHHH

Q Fri_Aug_01_22: 581 EALEAFAERVASPAGDRAAHEFLFREVVANDS-GALLGAPFGCRRQR----------------------GPRLGHYAAFA 637 (688)

Q Consensus 581 ~~~~~i~e~~a~~~~~~~~~~~~~~~~va~~~-~lL~~~~~~~~~~~----------------------~p~~~~f~AFA 637 (688)

.-++.+.+ .+..+-..... .+-.++|-- +.+-..... . .|.+.+.|++-

T Consensus 362 ~~i~~l~~-~R~~~k~~~~~---~~Kl~~NS~YG~~g~~~~~----~~~~~~~~~~~~~~~~~~~~~~~~p~~~~ia~~i 433 (575)

T 2py5_A 362 DFIDKWTY-IKTTSEGAIKQ---LAKLMLNSLYGKFASNPDV----TGKVPYLKENGALGFRLGEEETKDPVYTPMGVFI 433 (575)

T ss_dssp HHHHHHHH-HHHHCCHHHHH---HHHHHHHSSHHHHTCCSBC----CBEEEEECTTSSEEEEECCCCBCCCCCHHHHHHH

T ss_pred HHHHHHHH-HhhhcCHHHHH---HHHHHHhhhHhhhccCccc----ceeEEEecCcceEEEecCcccccCCcchhhhhHH

Q ss_pred HHHHHHHHHHHHHHhhhhhhhhhhhcccceEEEec

Q Fri_Aug_01_22: 638 RGYARVAAHELMRRLDNEYCPRVVSSYTSSRVFVR 672 (688)

Q Consensus 638 aSYCR~~iH~LI~~IDshy~g~~V~~hny~~i~vr 672 (688)

-+|.|..+++.|+. +|.-|+--.++-|++.

T Consensus 434 t~~~R~~l~~~~~~-----~~~~viY~DTDSi~~~ 463 (575)

T 2py5_A 434 TAWARYTTITAAQA-----CYDRIIYCDTDSIHLT 463 (575)

T ss_dssp HHHHHHHHHHHHHH-----TTTTEEEEETTEEEEE

T ss_pred HHhHHHHHHHHHHh-----ccCcEEEecCcceEEc
