# Supplementary material for: Chordopoxvirus protein F12 implicated in enveloped virion morphogenesis is an inactivated DNA polymerase
Source: Biol Direct. 2014 Nov 6;9:22. doi: 10.1186/1745-6150-9-22 (PMC4304020; doi:10.1186/1745-6150-9-22)
Supplement: Additional file 2 — Phyre 2 results for VACV F12. [file 1745-6150-9-22-S2.docx]

# Additional File 3

Phyre2 [[1](#_ENREF_1)] was used from the webserver at: <http://www.sbg.bio.ic.ac.uk/phyre2>.

Default parameters were used.

| PDB | Confidence | %id | Coverage (635) | Template Coverage | Species |
| --- | --- | --- | --- | --- | --- |
| 2ex3 | 98.8 | 13 | 223-604 | 7-449 (575) | *Bacteriophage phi29* |
| 2py5 | 98.3 | 10 | 426-608 | 216-253 (575) | *Bacteriophage phi29* |
| 2gv9 | 93.3 | 15 | 192-461 | 360-726 (1193) | *Simplex Herpes* |
| 1ih7 | 92.9 | 25 | 184-296 | 92-229 (903) | *Enterobacteria phage RB69* |
| 1s5j | 91 | 19 | 192-297 | 223-326 (847) | *Sulfolobus sulfataricus* |
| 1noy | 90.7 | 15 | 184-297 | 96-227 (388) | *Enterobacteria phage T4* |

Phyre2 reformatted results for the complete F12 sequence of VACV.

1. Kelley LA, Sternberg MJ: **Protein structure prediction on the Web: a case study using the Phyre server**. *Nat Protoc* 2009, **4**(3):363-371.
