# Supplementary material for: Chordopoxvirus protein F12 implicated in enveloped virion morphogenesis is an inactivated DNA polymerase
Source: Biol Direct. 2014 Nov 6;9:22. doi: 10.1186/1745-6150-9-22 (PMC4304020; doi:10.1186/1745-6150-9-22)
Supplement: Additional file 4 — TPRpred results for the relevant poxvirus proteins and kinesin light chains. [file 1745-6150-9-22-S4.docx]

# Additional File 4

# TPRpred results

TPRpred [[1](#_ENREF_1)] were used from the webserver at: http://toolkit.tuebingen.mpg.de/tprpred.

Default parameters were used, except the e-value which was increased to 0.1 to increase the search sensitivity.

## F12 Vaccinia virus

No significant hit detected.

## F12 homolog Nile crocodile virus

No significant hit detected.

## A36 Vaccinia

No significant hit detected.

## E2 Vaccinia

No significant hit detected.

## KLC1 Human

Per-protein P-value for being TPR : 4.0E-41

Probability for being TPR : 100.00%

Repeat Begin Alignment End P-value

-------------------------------------------------------------------

TPR 213 LRTLHNLVIQYASQGRYEVAVPLCKQALEDLEKT 246 5.4e-08

TPR 255 ATMLNILALVYRDQNKYKDAANLLNDALAIREKT 288 5.6e-08

TPR 297 AATLNNLAVLYGKRGKYKEAEPLCKRALEIREKV 330 6.5e-12

TPR 339 AKQLNNLALLCQNQGKYEEVEYYYQRALEIYQTK 372 6.3e-10

TPR 381 AKTKNNLASCYLKQGKFKQAETLYKEILTRAHER 414 1.4e-09

TPR 464 TTTLKNLGALYRRQGKFEAAETLEEAAMRSRKQG 497 1.4e-05

## KLC2 Human

Per-protein P-value for being TPR : 1.4E-36

Probability for being TPR : 100.00%

Repeat Begin Alignment End P-value

-------------------------------------------------------------------

TPR 198 LRTLHNLVIQYASQGRYEVAVPLCKQALEDLEKT 231 5.4e-08

TPR 240 ATMLNILALVYRDQNKYKEAAHLLNDALAIREKT 273 2.6e-08

TPR 282 AATLNNLAVLYGKRGKYKEAEPLCKRALEIREKV 315 6.5e-12

TPR 324 AKQLSNLALLCQNQGKAEEVEYYYRRALEIYATR 357 7.6e-08

TPR 366 AKTKNNLASCYLKQGKYQDAETLYKEILTRAHEK 399 1.4e-09

TPR 408 NKPIWMHAEEREESKDKRRDSAPYGEYGSWYKAC 441 9.0e-01

TPR 449 NTTLRSLGALYRRQGKLEAAHTLEDCASRNRKQG 482 2.3e-04

TPR 489 TKVVELLKDGSGRRGDRRSSRDMAGGAGPRSESD 522 2.4e-01

TPR 530 AEWNGDGSGSLRRSGSFGKLRDALRRSSEMLVKK 563 1.8e-02

1. Karpenahalli MR, Lupas AN, Soding J: **TPRpred: a tool for prediction of TPR-, PPR- and SEL1-like repeats from protein sequences**. *BMC Bioinformatics* 2007, **8**:2.
